# Foresight for the Fitness Sector: Results from a European Delphi Study and Its Relevance in the Time of COVID-19

**DOI:** 10.3390/ijerph17238941

**Published:** 2020-12-01

**Authors:** Louis Moustakas, Anna Szumilewicz, Xian Mayo, Elisabeth Thienemann, Andrew Grant

**Affiliations:** 1 Institute for European Sport Development and Leisure Studies, German Sport University, 50933 Cologne, Germany; 2 Faculty of Physical Culture, Gdansk University of Physical Education and Sport, 80-336 Gdansk, Poland; anna.szumilewicz@awf.gda.pl; 3 Observatory of Healthy and Active Living of Spain Active Foundation, Centre for Sport Studies, King Juan Carlos University, 28942 Madrid, Spain; xian.mayo@urjc.es; 4 Independent Scholar, 1000 Brussels, Belgium; elisabeth.thienemann@gmail.com; 5 Independent Consultant, Sunderland SR5 1SN, UK; andygrantconsultancy@gmail.com

**Keywords:** fitness, exercise, foresight, delphi study, Europe

## Abstract

The fitness sector is an essential player in the promotion of physical activity and healthy behaviour in Europe. However, the sector is confronted with numerous socio-demographic trends that will shape its ability to be financially successful and contribute to public health. The sector must understand current drivers of change and the skills its workforce needs to navigate them. As such, using the results of a 2019 Delphi Survey of over 50 fitness experts from 26 countries, we aim to define the drivers of change facing the sector and identify the skills needed by the fitness workforce to navigate these changes. We find that several technological, social, health and economic trends affect the sector. As a result, so-called soft skills such as communication or customer service, along with digital technology skills, are becoming increasingly important. There is also growing recognition that fitness professionals need to be trained to work with a number of special populations. Furthermore, we argue that many of the trends identified here—such as the increasing use of technology or the focus on individual customer needs—have been accelerated by the COVID-19 pandemic. We conclude by arguing that well-developed, pan-European qualifications are needed to address these common issues.

## 1. Introduction

Physical activity helps prevent and treat a plethora of non-communicable diseases while also improving mental health, quality of life, and well-being. Despite these well-known benefits, over the last decade in Europe, there has been a decrease in overall physical activity and a concomitant increase in obesity. Between 25 and 35% of the European population is physically inactive [1], and similar percentages are obese [2]. In its latest Global Action Plan, the World Health Organisation emphasised the importance of building active societies as a strategic objective to strengthen the promotion of physical activity. In this regard, supporting pre- and in-service training to increase knowledge and skills related to the fitness sector professionals, along with other health sector stakeholders, was proposed as a policy instrument to help reduce worldwide physical inactivity by 2030 [1]. Similarly, the Regional Office for Europe of the World Health Organization suggests that states should support membership in fitness centres as a way to promote physical activity amongst younger and more vulnerable demographic groups [2].

These facts reinforce the importance of providing high-quality, responsive fitness offers in a variety of communities and settings. To some extent, the fitness industry has attempted to address these needs and support the current public health agenda. For instance, the industry has increased the number of facilities available and has also promoted the development of recognised qualifications. As a result, over the last decade, fitness users have increased by 72%, and almost 10% of all European adults are users of fitness services [3]. Authors of previous reports have tried to analyse the ongoing professionalisation of the industry, pointing out areas where the workforce excel, what the emerging trends are, and where the industry should turn its focus [4]. In this sense, and despite this business growth, fitness employers face numerous challenges that impede service delivery. There are significant skill shortages and mismatches within the fitness workforce. Based on Cedefop’s skills online vacancy analysis tool, the European fitness sector alone is seeking to fill 115,000 vacant positions [5]. Employers also struggle to find staff with the right skill set to meet the industry’s evolving needs. One in five employers report difficulties recruiting the trainers they want to work in their clubs [6]. Furthermore, fitness employers must consider how their current and future workforce will need to evolve to support changing public health needs. In short, though the industry has been active in trying to sustain its growth and support public health, there remain significant present and future challenges.

It is against this backdrop that EuropeActive (EA)—the European body for the fitness industry—launched the Sector Skills Alliance (SSA) for Active Leisure [7] according to the Council of the European Union declaration on the European Alliance for Apprenticeships [8]. From the SSA, the Blueprint for Skills Cooperation and Employment in Active Leisure project emerged [9]. These initiatives aim at developing a sector skills strategy for the fitness sector in collaboration with several European organisations, representing employers and employees, national authorities, training providers, higher education, and research institutions, as well as other relevant sector stakeholders.

Building on findings from the Blueprint project [10], the following paper aims to answer two main questions: (1) what are the main drivers for change and development in the fitness sector by 2030 and how will this impact fitness services? and (2) what skills will fitness professionals need by 2030 in response to these drivers? Identifying these drivers and skill requirements not only allows the fitness industry to ensure its continued sustainability, but it also provides valuable insights for the sector to continue supporting the public health challenges described above. Based on the results of a European Delphi study of experts from across the fitness sector, our study aims to identify the drivers most likely to impact the fitness sector and map out the specific skills needed within the fitness workforce to address them. Furthermore, as this study was conducted in 2019, we will argue that the trends and skills identified remain highly relevant despite the massive global changes created by the COVID-19 pandemic.

## 2. Materials and Methods

### 2.1. Design

The Delphi method has traditionally been used for several purposes, including aggregating ideas, generating consensus and forecasting [11,12]. The current study is aligned with this latter purpose. As outlined above, our goal is to identify the main drivers for change and development in the fitness sector as well as the related skill requirements for fitness professionals. Here, we define fitness professionals as instructors, trainers, and other exercise specialists, qualified to deliver diverse, structured exercise programmes that help people of all ages and abilities to improve their fitness, and physical and mental health [4].

In the brainstorming phase, a focus group discussion allowed experts to elaborate on the influences affecting the fitness sector. Based on the qualitative results generated, we implemented two survey rounds for a broader panel of experts. Within this survey, experts were asked to establish preliminary priorities among the survey items. Specifically, respondents were asked to evaluate, on a four-point scale, if a particular trend or challenge was either significant (4 = Significant Impact to 1 = No Impact) or likely (4 = Extremely likely to 1 = Unlikely). After the initial survey round, a second survey was sent whereby each expert received a questionnaire with the priorities summarised from the previous round. This second survey allowed experts to confirm or revise their opinions. Typically, the literature on the Delphi method suggests that consensus is achieved somewhere between 70% and 80% [11,12,13]. Given this, here, a consensus was determined when at least 75% of respondents ranked an item as either extremely likely/likely or as having significant/moderate impact. Finally, to further validate findings, survey results were presented and discussed in a smaller face-to-face expert focus group.

Finally, it should be noted that our data was collected shortly before the COVID-19 pandemic. Nevertheless, we analyse them later in the context of the needs and changes in the fitness sector caused by the situation. Additionally, in November 2020, our outcomes were discussed by a broad group of experts during the 3rd Meeting of the Sector Skills Alliance in Active Leisure [14] and The 11th EuropeActive International Standards Meeting [15].

### 2.2. Sampling

Purposive and snowball sampling were used to identify participants. As the Delphi method relies more on group consensus building than on statistical power, we aimed to achieve at least the recommended minimum sample size of 20 [13]. Experts were selected based on their experience and engagement in the European fitness sectors, be it in terms of advocacy, management, implementation, or research. As such, experts could represent national fitness associations, fitness providers, governmental bodies, European organisations, or academic institutions. Thus, the experts possessed both a wealth of experience as well as the diverse set of perspectives needed to build industry-wide consensus. Ultimately, this range of experts enhanced the credibility and applicability of the results, increasing the overall reliability of the findings. Similarly, the inclusion of experts who have knowledge of the sector and the multiple rounds of data collection support the validity of the results [12].

We targeted experts for the brainstorming amongst participants of the Sector Skills Alliance (SSA) for Active Leisure meeting held on the margins of the 9th EA International (Fitness) Standards Meeting (ISM) in Warsaw, Poland, in 2018. We further identified experts based on references provided by staff at EuropeActive and partners of the Blueprint project, leading to a list of 152 experts. Prior to taking part in the study, we explained the purpose of the study and that participation in the process was voluntary. Furthermore, all responses have been anonymised for publication.

### 2.3. Data Collection

#### 2.3.1. Brainstorming

The brainstorming phase took place amongst members of SSA during the 2018 ISM in Warsaw, Poland, where a focus group discussion was held. The brainstorming looked to identify drivers of change in the fitness sector and how these drivers might impact the provision of services as well as employment in fitness. This discussion took place on 14 November 2018 with 17 participants and was jointly conducted by the fourth and fifth authors.

#### 2.3.2. Online Survey

The survey was sent out in two rounds, and each round was sent to the 152 identified experts. The first round was conducted between 19 February 2019 and 3 March 2019, leading to 54 replies (response rate of 35.5%). After completion of the first round, we reviewed the data and reformulated open-ended responses into statements for verification in the second round. The second round was held between 11 and 24 March 2019, generating 50 replies (response rate of 30.4%). Overall, the sampled experts included a broad range of relevant sub-fields and represented over 25 European countries. Most prominently, vocational training providers, government representatives, and employers represented more than half of responses. Furthermore, 72% of respondents reported having more than ten years of experience in the industry. The breakdown of the background and location of experts are presented in Table 1 and Table 2, respectively.

#### 2.3.3. Validation

In April 2019, we compiled data from the two survey rounds and sent a summary to 21 members of the Sector Skills Alliance for online review and feedback. Additionally, we further validated our findings through a supplementary focus group discussion on 27 June 2019 with 16 members of the Sector Skills Alliance. This discussion was jointly conducted by the first author and an external consultant.

### 2.4. Data Analysis

Qualitative (i.e., open-ended responses) generated throughout the Delphi survey process were analysed following a process of thematic analysis. This process helped us identify the main trends and issues that emerged throughout the open-ended responses and focus group discussions. We used the process of thematic analysis described by Braun and Clarke [16] to organise the data. This included becoming familiar with the data, searching for themes, reviewing potential themes, defining final themes, and writing the data. Quantitative, Likert scale data obtained from the Delphi surveys were entered into a tabular format, and descriptive statistics were generated.

## 3. Results

### 3.1. Main Drivers for Change and Development in the European Fitness Market Over the Next Decade

**Figure 1:**
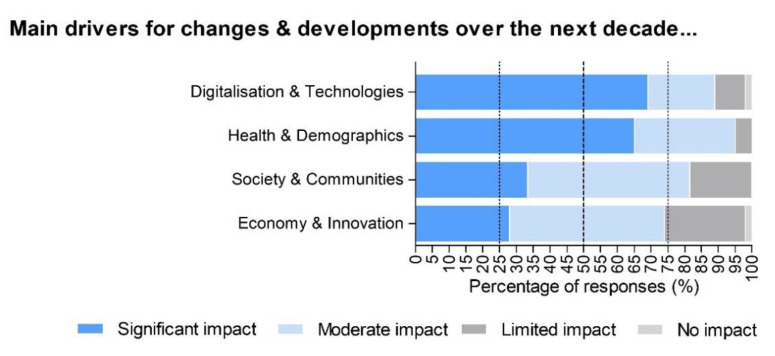
Level of impact of the main drivers for change and development over the next decade in percentages (%) by the experts (*n* = 50). In dark blue, significant impact. In light blue, moderate impact. In black grey, limited impact. In light grey, no impact.

As can be observed in Figure 1, 69% of the experts found that digitalisation and technologies will have a significant impact on the fitness sector over the next decade. Likewise, 65% of experts felt that health and demographics will have a significant impact on the industry by 2030. Other potentially significant drivers such as society and communities (48% of the experts) and economy and innovation (28% of the experts) were rated by the experts less highly in comparison.

### 3.2. Characteristics of Fitness Places Over the Next Decade

**Figure 2:**
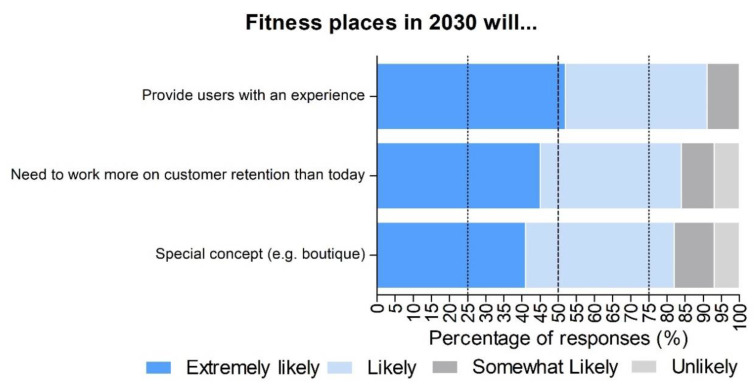
Levels of likeliness rated in percentages (%) by the experts (*n* = 50) about how fitness places will be by 2030. In dark blue, extremely likely. In light blue, likely. In black grey, somewhat likely. In light grey, unlikely.

Figure 2 shows the potential characteristics of fitness places and how likely they will be by 2030. In this regard, more than half of the experts (52%) perceived it as extremely likely that fitness places will provide an experience to the users. More specifically, this experience should be inherently social and support the creation of fitness communities. Following closely behind, 45% of the experts reported it as extremely likely that fitness centres will work more on customer retention than at present. Lastly, 41% of the experts reported that fitness places will adopt a special concept such as micro-gyms, boutiques, or high-tech gyms.

### 3.3. Skills of Fitness Professionals Over the Next Decade

**Figure 3:**
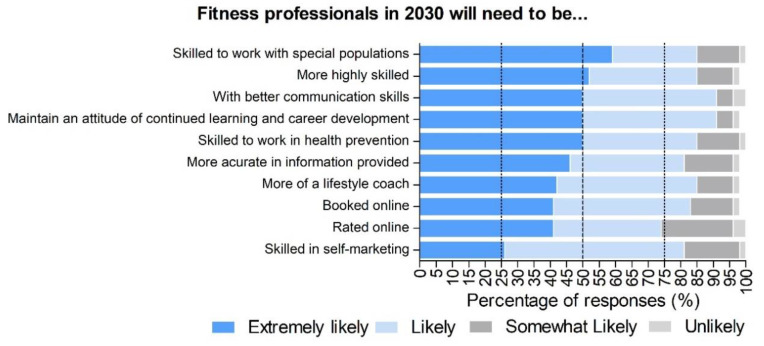
Levels of likeliness rated in percentages (%) by the experts (*n* = 50) about the required skills/characteristics of fitness professionals by 2030. In dark blue, extremely likely. In light blue, likely. In black grey, somewhat likely. In light grey, unlikely.

Figure 3 depicts how the experts rated the likeliness of several variables related to the future skills and requirements of fitness professionals. In this regard, the responses indicated that higher levels of agreement exist over two main issues. First, there was a broad agreement over the need for fitness professionals to work on a variety of health issues and with a range of populations. Items including skilled to work with special populations (e.g., older people, people with non-communicable diseases), skilled to work in health prevention, and being more of a lifestyle coach were rated as extremely likely by 59%, 50%, and 42% of the experts, respectively. Second, various soft skills were seen as increasingly required. Items including being highly skilled, having better communication skills, maintaining an attitude of continued learning and career development, and providing more accurate information, were rated as extremely likely by 52%, 50%, 50%, and 46% of the experts, respectively. Elsewhere, variables related to technology did not generate the same levels of consensus. Items such as being booked and rated online and skills connected with self-marketing were rated as extremely likely by 41%, 41%, and 26% of experts, respectively.

## 4. Discussion

Using the results of a 2019 Delphi Survey, our study aimed to define the drivers of change facing the fitness sector, understand how these drivers will influence fitness places, and outline the professional skills needed to navigate these changes. Thus, our work fits in with other research looking at trends in the industry. However, much of this other work focuses on a single group of respondents such as fitness trainers [17] or employers [6]. In that sense, our present study is unique as it generates findings and consensus across a broad range of experts, hence ensuring a wider variety of perspectives and increased validity.

First and foremost, our results highlight the need for the fitness sector and fitness professionals to be better aligned with the health sector to contribute to the emerging public health agenda. For instance, the importance of working with special populations (e.g., older people, people with non-communicable diseases), working in health prevention and being more of a lifestyle coach rated very highly in our study. This aligns strongly with other studies, both pre- and post-pandemic, that also found that skills relevant to disease prevention and health promotion are critical for fitness professionals [6,17,18]. Furthermore, the desire to improve the health of the clients and reduce non-communicable diseases are some of the primary motivators behind the work and further education of fitness professionals [19].

The continued development of links between the fitness industry and the health sector is not only relevant in light of the challenges posed by obesity or physical inactivity, but emerging research suggests that the fitness sector can play a critical role in mitigating the effects of COVID-19. Physical activity can play a significant role in reducing COVID-related mortality and will have a crucial role in recovering health lost during the pandemic. Physical fitness enhances the immune system [20] and helps reduce obesity, which is a predictor of mortality amongst COVID patients [21]. In addition, physical activity can help to recover from the effects of severe lockdowns and restrictions on physical and mental health [22]. In any event, the survey and focus group results highlight the need for the fitness sector to work more closely with the health sector to support primary prevention of non-communicable diseases and supporting behaviours change for healthy lifestyles more generally. Though in the past, there have been medical referral schemes connected to the fitness sector, this referral was specialized more in clinical exercise interventions helping to manage secondary and tertiary prevention (i.e., preventing a disease from getting worse or managing an existing disease) [23]. We must also note that medical professionals must likewise become better equipped to interact with the fitness sector. Based on recent studies, general practitioners show very low levels of knowledge about physical activity guidelines and exercise promotion in primary health care is very rare [24,25,26]. According to Füzéki et al. [24] “strengthening the topics of physical activity and health and physical activity counselling in medical curriculum is strongly recommended” as it would prepare health care providers for effective collaboration with fitness professionals to support increased physical activity levels.

Another group of variables increasingly needed by fitness professionals are related with the soft skills (i.e., personal transversal competencies such as social aptitudes, communication, teamwork, and other personality traits) that improve the provision of services and products. In our study, being highly skilled, having better communication skills, maintaining an attitude of continued learning and career development, and providing more accurate information were rated as extremely likely requirements for fitness professionals by around 46–52% of the respondents. Yet, though these skills are seen as more and more essential, research has suggested that fitness professionals lack many of the required communication, social, and counselling skills [19,27]. This mirrors concerns from employers in other industries [28], and most fitness professionals need additional training when starting a job. This reflects the changing nature of the fitness profession, whereby how to communicate and engage with clients is now a cornerstone of the provision of services. Indeed, most fitness professionals possess entry-level (e.g., EQF level 3 or 4) instructor or personal trainer qualifications [29]. Educational standards for these levels contain learning outcomes related to communication, client motivation, and counselling [30]. The low level of soft skills among fitness professionals, observed by other authors, may result from the way their education and training is carried out and how the required competencies are verified. Indeed, it is much more challenging to assess the achievement of soft skills compared to technical skills. This conclusion has been incorporated into work on the development of learning outcomes assessment strategy for fitness professionals under the Blueprint project [9]. Yet the need for interpersonal skills and continued learning are embedded in the ever-changing notions of the fitness profession. Fitness trainers now routinely take on a multitude of roles, including those of teacher, trainer, counsellor, coach, supervisor, supporter, nutritionist, life management advisor, weight controller, or even personal life consultant [31,32,33]. In addition, fitness professionals will need to be given the skills and knowledge to deliver services in a variety of new settings. At present, fitness centres are predominantly large-scale facilities solely dedicated to the provision of exercise services. Moving forward, numerous new fitness concepts are emerging, including integrated wellness offerings, outdoor gyms, smaller boutique gyms, and even gyms within retail locations [17,34,35]. Technology also directly impacts the scope and nature of the skills required. As noted as early as 1960 by Kerr [36], technology directly influences skill requirements. Today, institutions must therefore find a balance between physical workplace demands and the skill sets essential to meeting current technological needs.

While technology was reported in this study and preceding reports as a critical driver for change in the fitness sector, in our analysis, technology-related skills were rated less highly compared to other skills for fitness professionals (i.e., being booked and rated online or skills related with marketing). This is in line with a previous report where technology-related trends were rated less highly compared to other issues [17]. However, almost 80% of owners, operators, and gym managers report that online training and the use of technology are emerging trends in the European region [6]. In other words, though technology is perceived as a critical driver, it is not entirely clear to fitness experts exactly how that will translate to fitness places or the skills of fitness professionals.

Nonetheless, based on developments in the broader health sector and the impact of the COVID-19 pandemic, we can anticipate some of the directions technology might take within the fitness sector. Experts from the World Health Organization noted that digital health, or the use of digital technologies for health, “has become a salient field of practice for employing routine and innovative forms of information and communications technology to address health needs” [37]. Related to this is the concept of mobile health (mHealth), defined as “the use of mobile wireless technologies for health” [37]. In the current landscape, we can already see manifestations of “digital fitness” or “mFitness”. For instance, individuals can continuously monitor their physical activity with the use of wearable technology [38]. Elsewhere, specialised applications provide quick feedback on the implementation of the prescribed exercise programme and may support the maintenance of client motivation [39].

The COVID-19 pandemic has further focused the industry’s attention towards providing digital or mobile services. The closure of fitness clubs and the compulsory limitation of direct social contact resulted in many people experiencing online home fitness for the first time. Consequently, during lockdown, many exercise professionals and gyms began to provide their services exclusively online. The technological drivers of change identified within our survey remain relevant but have indeed been greatly accelerated. Further, this technological shift is likely to continue after the pandemic as the benefits and risks of online training become ever better understood [40,41,42].

In response to the growing need for digital fitness offerings, EuropeActive has been developing a supplementary qualification concerning the online provision of fitness services (9). It includes the professional competencies necessary for fitness professionals to be able to carry out their professional tasks safely and effectively while using online tools [43]. Here, online fitness services should be understood as something much broader than just conducting live exercise sessions via online technology. It includes all the stages related to guiding and coaching clients to positive lifestyle behavioural change and integrating healthy behaviours into daily routines [44].

Similar qualifications for various fitness professionals, aligned with the European Qualification Framework (EQF) and its associated recommendations, have been developed over the last many years to address many of the special, or clinical, populations identified above. At present, specialised qualifications related to active ageing, diabetes, and pre- and post-natal exercise are formally in place [45]. Ultimately, these current and future qualifications support the delivery of improved, increasingly relevant professional skills.

However, there remain substantial challenges in the delivery and recognition of these qualifications. Not all qualifications are delivered in all countries. Further, often, qualifications that are aligned to the EQF and recognised on the National Qualification Framework (NQF) of one country are not formally recognised in other EU member states. In turn, these factors conspire to limit the development and mobility of fitness professionals—which then further impedes the sector’s ability to address the drivers of change identified here. As a result, this provokes a shortage of qualified professionals in the sector and limits individual career development. In response, we argue that more European coordination, development, and mutual recognition of qualifications are needed to help the fitness sector become a holistic, well-developed sector able to support the current public health agenda. To that end, both the European Commission and individual countries have a responsibility to support the development of relevant qualifications and ensure their recognition across the continent. As our study makes clear, there is consensus about the drivers of change and skills required across a broad thematic and geographic range of experts. Though we do not wish to dismiss country-specific needs or realities, our results make clear that many pan-European challenges would benefit from pan-European solutions.

Finally, we recognize that there are important financial implications involved in the development and delivery of qualifications, as well as in the remuneration of an increasingly educated and professionalized workforce. It is certainly beyond the purview of our paper to engage in a comprehensive cost-benefit analysis. However, it is clear to us that current difficulties in attracting and retaining qualified staff as well as the importance of client retention would more than likely justify these investments. Staff turnover is a high cost for businesses, and recent research in the fitness sector suggests that more educated staff can support client retention [46,47]. If the fitness industry does not upgrade and update its qualifications to meet current challenges and ensures a satisfied, well-paid workforce, the challenges, and threats, posed by lacklustre staff and client retention will remain. In addition, investment in high-quality exercise specialists and the promotion of physical activity can potentially lower the social costs associated with treating diseases caused by physical inactivity. This issue is still being analysed by other authors [23].

As the first Delphi study on foresight for the fitness sector, our work has its limitations. For instance, some factors, based on the brainstorming phase, have been combined, like “innovation and economy” or “health and demographics”. In hindsight, analysing them separately would have provided more information on the main drivers of development in the fitness sector. There is also a need to integrate fitness clients into such studies, as these end-users are often absent in research on the sector. Similarly, many forecasting studies are predicated on pan-European perspectives. However, there remains room for nationally-focused, comparative research that looks to identify commonalities and differences across individual European states. Ultimately, such multi-directional analysis would allow for a better understanding of the sector’s needs and more effective preparation against upcoming changes.

## 5. Conclusions

Building on the results of a 2019 Delphi Survey conducted in the context of EuropeActive’s Blueprint project, our study aimed to define the drivers of change facing the fitness sector, understand how these drivers will influence fitness places, and outline the professional skills needed to navigate these changes. Ultimately, we find that technology, healthcare needs, and customer retention are critical drivers of change in the fitness industry. In response to this, fitness professionals must improve both their professional skills, especially as they relate to service provision for special populations, as well as their soft skills.

Though the global landscape has changed significantly since this study was conducted due to the COVID-19 pandemic, we argue that the results here are as relevant as ever. The ability to engage with technology and to have an understanding of specific health-related issues have only come into sharper focus due to the pandemic and its related social changes.

Moving forward, it will be imperative for the fitness sector to continue developing pan-European solutions and qualifications to address these continent-wide trends and challenges. Likewise, government agencies must redouble their efforts to recognise such qualifications both within their countries and from other European countries.

## Figures and Tables

**Table 1:** Background of Experts.

	Round 1 (*n* = 54)		Round 2 (*n* = 50)	
Description	Number	Per Cent	Number	Per Cent
Vocational Training Provider	15	27.78%	7	14.00%
Government/Government Agency/NGO/EU institution (EU/national/regional/local)	12	22.22%	11	22.00%
Operator (employer)	10	18.52%	10	20.00%
Other (e.g., consultant, federation)	9	16.67%	10	20.00%
Researcher/higher education institute/university	5	9.26%	8	16.00%
Other fitness professional	2	3.70%	2	4.00%
Personal Trainer or higher-skilled fitness professional	1	1.85%	1	2.00%
Public Employment Service (National/EURES)	0	0.00%	1	2.00%

**Table 2:** Location of Experts.

Country	Round 1 (*n* = 54)	Round 2 (*n* = 50)
Belgium	5	5
Bulgaria	1	0
Croatia	1	0
Cyprus	1	0
Czech Republic	0	1
Denmark	1	0
Finland	5	5
France	1	2
Germany	4	4
Greece	2	2
Hungary	1	1
Iceland	1	1
Ireland	1	3
Italy	4	2
Malta	1	1
Moldova	1	0
Other	0	2
Poland	4	1
Portugal	1	2
Russia	2	0
Slovenia	0	1
Spain	5	6
Sweden	1	3
The Netherlands	7	4
Turkey	1	1
United Kingdom	3	3
